# Galectin-13 and Laeverin Levels Interfere with Human Fetoplacental Growth

**DOI:** 10.3390/ijms25126347

**Published:** 2024-06-08

**Authors:** Márió Vincze, János Sikovanyecz, Imre Földesi, Andrea Surányi, Szabolcs Várbíró, Gábor Németh, János Sikovanyecz, Zoltan Kozinszky

**Affiliations:** 1 Department of Obstetrics and Gynecology, University of Szeged, H-6725 Szeged, Hungary; vincze.mario92@gmail.com (M.V.); janossikovanyecz@gmail.com (J.S.J.); gaspar-suranyi.andrea@med.u-szeged.hu (A.S.); varbiroszabolcs@gmail.com (S.V.); nemeth.gabor@med.u-szeged.hu (G.N.); drsikovanyecz@gmail.com (J.S.); 2 Department of Laboratory Medicine, University of Szeged, H-6720 Szeged, Hungary; foldesi.imre@med.u-szeged.hu; 3 Capio Specialized Center for Gynecology, Solna, 182 88 Stockholm, Sweden

**Keywords:** Gal-13, laeverin, human, serum, amniotic fluid, fetus, placenta, sonography

## Abstract

Galectin-13 (Gal-13) is predominantly produced by the syncytiotrophoblast, while laeverin is expressed on the outgrowing extravillous trophoblast, and both are thought to be biomarkers of preeclampsia. The aim of this study was to assess the correlation between concentrations of Gal-13 and laeverin measured in maternal serum and amniotic fluid at 16–22 weeks of gestation and the sonographic assessment of the fetoplacental measurements. Fetal biometric data and placental volume and perfusion indices were measured in 62 singleton pregnancies. Serum and amniotic levels of Gal-13 and laeverin levels were measured using a sandwich ELISA. Both amniotic fluid and serum Gal-13 levels expressed a negative correlation to the plasma laeverin level in mid-pregnancy. Serum laeverin level correlated positively with the gestational length at delivery (β = 0.39, *p* < 0.05), while the amniotic laeverin level correlated well with the abdominal circumference of the fetus (β = 0.44, *p* < 0.05). Furthermore, laeverin level in the amnion correlated positively with the estimated fetal weight (β = 0.48, *p* < 0.05) and with the placental volume (β = 0.32, *p* < 0.05). Logistic regression analyses revealed that a higher circulating Gal-13 level represents a slightly significant risk factor (OR: 1.01) for hypertension-related diseases during pregnancy. It is a novelty that laeverin can be detected in the amniotic fluid, and amnion laeverin concentration represents a potential biomarker of fetoplacental growth.

## 1. Introduction

The placenta expresses immune modulators which interact with maternal cells, providing predominantly an anti-inflammatory status in the second trimester drifting to a physiologic pro-inflammatory process before delivery [1]. Galectins are glycan-binding proteins belonging to the lectin subfamily. Galectin-13 (Gal-13) is expressed uniquely by the syncytiotrophoblast (STB) in the placenta. The function of Gal-13 encompasses remodelling of the spiral arteries in the uterus, presenting immune tolerance of the mother to the offspring [2,3]. Gal-13 stimulates the enlargement of uterine vessels during pregnancy via endothelial nitric oxide synthase and prostaglandin signaling pathways [4]. Gal-13 binds to the extracellular matrix, structurally stabilizing the expansion of the uterine arteries and veins during pregnancy [5]. Gal-13 is an anti-inflammatory molecule and is 7.16-fold upregulated during pregnancy [6]. Gal-13 sustains the survival of neutrophils, stimulating them to release reactive oxygen species, hepatocyte growth factor, and matrix metalloproteinase 9, providing materno-fetal immune tolerance [7]. Gal-13 facilitates extravasation of the T cells to the decidua [3] and creates necrotic zones to separate immune cells from the decidual cells, allowing trophoblast invasion and vascular remodeling [8]. Gal-13 is secreted into the maternal circulation from as early a stage of pregnancy as the fifth gestational week [9]. Increasing evidence shows the importance of Gal-13 in predicting preeclampsia, fetal growth restriction, and miscarriage, which is associated with an impaired uterine vascular adaptation [10].

It has been shown that laeverin expression is localized on the EVTs that invade decidual tissues and infiltrate the outer layer of the chorion leave, but not the amniotic epithelium [11]. Laeverin, also called aminopeptidase-Q, may play a regulatory role in the EVT migration to the decidua and the myometrium and in the reconstruction of spiral arteries. Laeverin on the cell surface of the EVTs exhibits cleaving activity on a wide range of substrates that promote angiogenesis (endokinin-C) or degrade antimigratory factors like kisspeptin-10 [1,12]. Cell-surface laeverin alters the cell surface receptor patterns (cell adhesion molecules) for matrix proteins, i.e., integrins favouring invasion. Furthermore, laeverin is one of the key molecules that mediate the communication between EVT and natural killer (NK) cells, monocytes/macrophages in the endometrium, and creates an immunosuppressive environment [13,14,15,16].

An increasing amount of literature is reporting on the potential role of laeverin in the pathomechanisms of preeclampsia, since preeclamptic trophoblasts overproduce laeverin [17,18,19] and ectopic intracellular expression of the protein [20] could be observed in preeclamptic placenta. Laeverin can be detected in the maternal serum [19,21] since EVT releases it into the maternal circulation. A lower circulating level might be a prognosticator of preeclampsia since the abundant laeverin is entrapped in the placenta [19], a disease with impaired uterine vascularity and placentation. Overexpression of laeverin disrupts the migratory capability of EVTs, resulting in shallow invasion and dysfunctional placental impairment [17,19,20].

In this study, we aimed to (1) evaluate the correlation between the serum and amniotic levels of laeverin and Gal-13, (2) determine whether laeverin is detectable in the amniotic fluid, and (3) assess the correlation between the amniotic and serum laeverin levels and fetoplacental growth measured by ultrasound.

## 2. Results

The phenotype of the sample is presented in Table 1. Predominantly, the women included in the study were older, overweight, and one third had no previous delivery. Typically, the women delivered at full term with normal neonatal weight (77.4%) except for two cases of premature births (3.2%). The sonographic characteristics of the fetuses and placentae are demonstrated in Table 2. The mean gestational age at the time of amniocentesis was 18.2 weeks.

**Figure 1:**
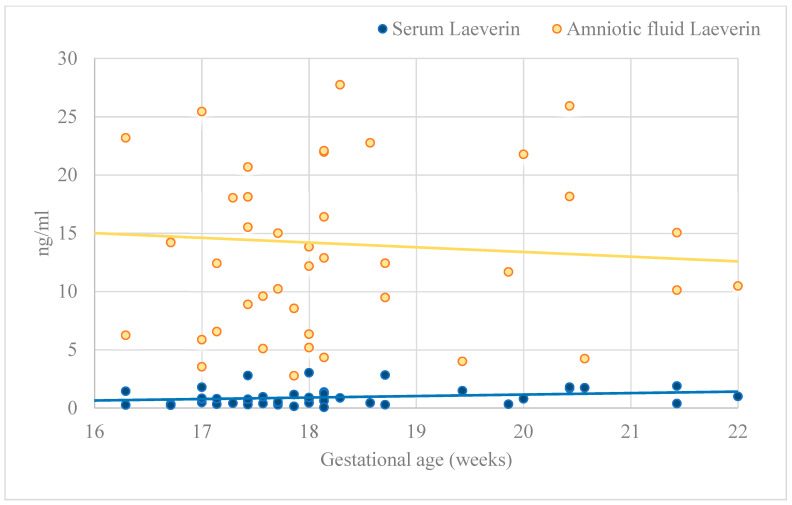
The secretion of laeverin in maternal serum and amniotic fluid.

Table 3 gives an overview of the placenta-derived factor levels in the body fluids. The mean serum Gal-13 concentration was 204.23 pg/mL, but its amniotic fluid concentration was lower with a magnitude (mean: 8.68 pg/mL). Conversely, the circulating laeverin concentration (mean 0.93 ng/mL) was much less in dimension than that in the amniotic fluid (mean: 14.11 ng/mL). The maternal serum laeverin level showed a negative correlation with the level of Gal-13 both in the serum (β = −0.38, 95% CI = −0.01–−0.01, *p* < 0.05) and in the amniotic fluid (β = −0.32, 95% CI = −0.04–−0.01, *p* < 0.05) in univariate analyses. Figure 1 demonstrates the laeverin levels in the body fluids according to gestational age. The laeverin levels were steady during this gestational period (*p* > 0.05). Mothers bearing female fetuses were exposed to almost double as high Gal-13 serum concentrations as compared to counterparts having male children (*p* = 0.039). More gender-related disparities were not observed in the samples.

Table 4 displays the correlation between the levels of laeverin with the fetoplacental biometrics assessed by sonography. Serum laeverin levels negatively correlated with maternal age (β = −0.34, 95% CI = −0.06–−0.01, *p* < 0.05) and positively with gestational age at birth (β = 0.41, 95% CI = 0.01–0.07, *p* < 0.05). Among fetal scanning parameters, a significant inverse correlation was detected between head circumference in percentile (β = −0.32, 95% CI = −0.01–−0.01, *p* < 0.05) and circulating laeverin level, and a strong positive interrelation between laeverin level in amniotic fluid and abdominal circumference (β = 0.55, 95% CI = 0.02–0.56, *p* < 0.05) and fetal weight (β = 0.48, 95% CI = 0.01–0.68, *p* < 0.05). Placental volume measurement was associated with the laeverin level in the amniotic fluid (β = 0.32, 95% CI = 0.01–0.46, *p* < 0.05) (Table 5).

Interrelation between the serum and amniotic fluid level of Gal-13 and the fetoplacental growth variables was published previously by our research group on a different sample with the same inclusion and exclusion criteria [22], and we have similar results in this study as well. Briefly, a significant negative correlation was observed between the Gal-13 levels in the amniotic fluid and neonatal birthweight (β = −0.26, 95% CI = −0.01–0.01, *p* < 0.05), and gestational length at delivery (β = −0.44, 95% CI = −0.66–−0.17, *p* < 0.05). The abdominal circumference of the fetus exhibited significant correlation to the Gal-13 levels measured in the amniotic fluid (β = 0.39, 95% CI = 0.04–0.57, *p* < 0.05), which was proved by multiple linear regression analysis (β = 0.60, 95% CI = 0.13–1.06, *p* < 0.05).

Logistic regression did not show any prognosticating effect of laeverin levels for GDM, hypertension, or any fetal weight deviation during gestation. Higher-serum Gal-13 levels presented a minimally increased odds for hypertension (*p* = 0.046, OR: 1.01, 95% CI: 1.00–1.03), but not for diabetes during gestation (*p* = 0.052, OR: 1.00, 95% CI: 1.00–1.03). No differential serum Gal-13 levels were found in pregnancies complicated with a large or small neonatal weight, and with appropriate weight.

## 3. Discussion

The principal finding of this study is that laeverin is detectable in amniotic fluid during mid-pregnancy obtained from amniocentesis. This is somehow in contrast with the results of Fujiwara et al. [11], who found that laeverin is expressed on the cell membrane of the EVTs in the chorionic villi and in the outer layer of the chorion laeve that surrounds the amniotic membrane, but the authors did not verify its secretion from the amniotic epithelium. This contrast might be explained by the fact that the other research group collected samples from placentae and fetal membranes at term, while our study group consisted of pregnant women during mid-pregnancy. The importance of laeverin decreases with advancing gestation, reflected by a decreasing serum level in the second half of pregnancy [19]. Since laeverin is a peptide, it cannot diffuse through a cell membrane and may be expressed into the amniotic cavity during gestation, but not towards term. One can speculate that EVTs may infiltrate into the amniotic cavity through the amniotic membrane, or that the amniotic epithelium cells release laeverin itself. Another study proved that laeverin is expressed not only in EVTs but also in STB and CTB [20,23], but this fact does not explain our interesting finding that placenta cells shed laeverin not only into the maternal and fetal circulation [19] but into the amniotic fluid as well.

By contrast, Gal-13 has previously been proved to be detectable in the amniotic fluid [24]; however, the exact mechanism of Gal-13 excretion into the amniotic cavity has not been described. Nonetheless, EVTs in the spiral arteries release laeverin into the maternal circulation. Human EVT invasion is regulated spatiotemporally, a process that occurs in the decidua in early pregnancy. Subsequently, EVT migrates to the spiral arteries and both processes are stimulated by angiogenic factors that interact with immune cells [1,14,16,25,26]. Laeverin is a membrane-bound protein exerting a peptidase effect [11], while Gal-13 modulates cell-signaling, cell adhesion interactions [4,5,7]. Interestingly, we observed that the levels of Gal-13 exhibit negative correlation with the laeverin levels in the maternal circulation. These two factors partially promote the same cellular and molecular differentiations. Gal-13 has a slightly decreasing tendency [22] during this period of pregnancy, while the maternal serum laeverin level is unchanged. This is somehow contradictory because the volume of the placenta expands in the mid-trimester following a linearly increasing curve [27], but these developmental processes are under the control of a network of various factors interacting with each other.

It is also contradictory that the serum level of laeverin was 0.93 ± 0.74 ng/mL up to 22 weeks of gestation in our study, whereas laeverin levels were higher in another study (median: 6.9 ng/mL at the 22nd gestational weeks) [19]. Furthermore, leverin concentration ranged in the serum between 41 and 393 ng/mL at a dilution of 1:50, during 8–14 weeks [21], which is also in contrast to the finding of much lower protein levels in our sample and in a previous study [19]. A possible reason for the discordance with other studies could be the use of different population and minor alterations among measurement kits [19,21]. The evaluation of serum laeverin levels was based on optical density measurements [19] or dilution series assessments [21] using a distinctly different assay than that utilized by our research group, although the kit analytes might be the same. The characteristics of the kits are not the same, e.g., intra- and inter-assay coefficient of variation, sensitivity [19,21]. Commercial kits detect a wide range of amino acid sequences of laeverin (818–905) capturing all types of isoforms of the protein (113 kDa, 130 kDA, 160 kDa and 231 kDa) [26]. Another study did not find a significant difference in the serum laeverin levels between the PE and the reference group, with an increasing tendency during the first trimester in both groups [21]. Notably, the laeverin levels decrease from the mid-trimester to term, which may reflect the declining physiological role of the laeverin in the third trimester [19]. The high levels of amniotic fluid laeverin (14.11 ± 9.18 ng/mL) in our study in the second trimester overlaps with the migration of EVTs in the decidua and the reconstruction of the walls of the spiral artery. However, the amniotic fluid does not communicate with the chorion, decidua, or maternal placental vessels, where laeverin exerts its effect [28]. One of the possible explanations may be that EVT produces laeverin both for maternal serum and amniotic fluid, although laeverin levels in different body fluids do not correlate to each other.

Furthermore, this is the first study to investigate the correlation of plasma and amniotic laeverin levels and sonographic parameters in pregnancies in mid-trimester. The maternal laeverin serum level corresponds well with the head circumference percentile of the fetus. It is of importance that laeverin measured in the amniotic fluid correlates strongly positively with the abdominal circumference, the estimated fetal weight of the offspring, and the placental volume. The reason for this is unclear; however, the larger placenta and fetal membranes may expand as gestation advances, and parallelly, the level of laeverin in the amniotic fluid also increases. Nonetheless, the placenta is the largest source of laeverin released into the fetal capillaries in the placenta [19] with no significant production or metabolism by the fetus. 

The present study showed that a higher serum Gal-13 in mid-pregnancy may prognosticate hypertension-related diseases, but not diabetes occurring as a late complication during gestation. Controversially, previous studies described that a significantly lower serum Gal-13 level in the first trimester is associated with the development of early- and late-onset preeclampsia later [8,29,30,31], relating to inadequate placentation. Gal-13 is a carbohydrate-binding protein and a member of the galectin group involved in placental implantation, uterine artery remodeling, and physiological inflammatory processes. Restructuring of spiral arteries co-occurs with the expansion of trophoblasts secreting Gal-13 protein with high intensity in the gestational interval of 16–22 weeks. Contrarily, a higher-serum Gal-13 predicts preeclampsia in our sample, which might be explained by the wide exclusion and inclusion criteria of the pregnant women in our study.

Impaired placentation is a primary cause of preeclampsia, and it is possible that reduced trophoblast volume could be one of the causes of lower-serum Gal-13 at 16–22 weeks of gestation in the women who later developed hypertension-related diseases. This is despite the comparable placental volume in the second trimester between pregnant women complicated by hypertensive disorders and unaffected women [32], and a decreased placental volume is only characteristic for preeclamptic pregnancies at term [33]. 

The role of Gal-13 in GDM has scarcely been investigated. Our study did not verify serum Gal-13 level as a useful screening modality for GDM. According to a previous study [34], a lower serum level in the third trimester and reduced placental expression of Gal-13 is associated with GDM, which is in contrast with our corresponding finding. Contrarily, Gal-13 serum levels did not differ between healthy pregnant women and women with GDM, but our result was almost significant (*p* = 0.052), and the sampling was in mid-gestation in our study but later in the other study [34]. Moreover, our subgroup analyses were underpowered to demonstrate significant differences regarding pregnancy complications. Further, the present study confirmed our previous results [22] on amniotic Gal-13 level exerting a predicting capability for gestational length at delivery and birthweight. Furthermore, the negative association between Gal-13 level in the amniotic fluid and fetal abdominal size is in correlation with our previous results. Additionally, we evaluated that laeverin serum and amniotic fluid levels do not indicate hypertension-related pathologies, GDM, or fetal weight anomalies as compared to uncomplicated pregnancies. 

This study has some limitations. Amniocentesis is usually performed to diagnose or exclude fetal aneuploidy or other fetal genetic risks. In molecular studies on samples obtained by means of amniocentesis, predominantly high-risk pregnancies are enrolled in molecular studies on amniotic fluid. The subjects in our study are older and have higher weight as compared to the national average reference age at delivery and pre-gravid weight in Hungary [35]. Another limitation is the small sample size, also because of the nature of the study sample and extensive exclusion criteria. Further, we were able to include only a small number of samples from women with pregnancy complications. Hence, the validation of the results of the present study requires a larger sample, and preferably first- and third-trimester pregnancies should also be included.

In conclusion, our study showed that although laeverin is produced by the EVTs, it can be detected in the amniotic fluid and its amniotic release remains elusive. The maternal serum laeverin levels are unchanged during mid-pregnancy. Amniotic laeverin levels correlate well with fetal size and placental volume. Women who later developed hypertensive disorder had slightly elevated levels of Gal-13 compared to healthy controls at 16–22 weeks of gestation in our cohort. Further research is needed to investigate the potential role of laeverin as a predictive biomarker of gestational diseases.

## 4. Materials and Methods

### 4.1. Study Design

A prospective, cross-sectional cohort study was performed in pregnant women having amniocentesis at the Department of Obstetrics and Gynecology, University of Szeged, Hungary between May 2022 and September 2022. We used the same inclusion and exclusion criteria for our present study population as earlier described [22]. During the study period, all women with singleton pregnancies with an increased risk of chromosomal abnormality, in which amniocentesis (AC) was performed between 16 + 0 and 22 + 0 weeks of gestation, were recruited into our study. The indications for AC were increased nuchal translucency (NT) at first trimester scan (≥2 MoM for gestational age) (n = 5), chromosome aberration or gene disorder concerning the previous pregnancy (n = 21), and advanced maternal age (≥35 years) (n = 36).

Exclusion criteria of the study were identified as follows: multiple pregnancies; fetal or neonatal structural or genetic anomaly; improper localization of the placenta for sonography (placenta praevia, posterior placenta); pathological placentation (placenta accreta spectrum); self-reported drug, alcohol, caffeine, or nicotine abuse or exposure to circulatory medication (oxerutins, calcium dobesilate); and systemic disease (e.g., essential hypertension, any type of pregestational diabetes mellitus, autoimmune disease, vasculitis, hemophylia, thrombophylia, chronic infections).

The following complications were registered in our sample: GDM (n = 11), hypertension-related diseases (n = 10), fetal growth restriction (FGR) at delivery (n = 4), large for gestational age at delivery (n = 9), premature birth (n = 2). A verbal and written explanation of the study was given to all the participants, and written informed consent was provided by all those who agreed to participate. Hence, our study population comprised 62 patients following amniocentesis. Clinical and anamnestic data were collected from the patients’ medical records.

The study protocol was approved by the Clinical Research Ethics Committee of the University of Szeged (date of approval: 10 February 2017, reference number: SZTE 09/2017). The study was carried out according to principles of the Declaration of Helsinki and its later amendments or comparable ethical standards.

### 4.2. Conventional 2-Dimensional (2-D) Sonographic Examinations

All the pregnancies were dated by using the measurement of crown–rump length (CRL) at nuchal screening. NT and anatomic assessment between 11 + 0 and 13 + 6 weeks were performed by utilizing conventional methods. Ultrasound examination took place before measuring AC to determine the number of fetuses, fetal biometry, fetal anomalies, placental location, and amount of amniotic fluid. Fetal weight was estimated according to the method of Hadlock et al. [36] after measuring the necessary sonographic parameters (biparietal diameter, head circumference, abdominal circumference, and femur length). The estimated fetal weight percentile was calculated according to local standards [37]. The ultrasound investigations were conducted by J.S. and A.S.

### 4.3. Volume Acquisition

The images used for the determination of placental volume and 3-dimensional Power Doppler (3-DPD) indices were acquired at the time of visit. All 3-D scans were performed with an A. S. Voluson 730 Expert ultrasound machine (GE Medical Systems, Kretztechnik GmbH & Co. OHG, Tiefenbach, Austria) equipped with a multifrequency probe (2–5 MHz). Each sample was examined using 3D rendering mode, in which the colour and gray value information was processed and combined to give a 3D image (mode cent; smooth: 4/5; FRQ: low; quality: 16; density: 6; enhance: 16; balance: 150; filter: 2; actual power: 2 dB; pulse repetition frequency: 0.9). We used fast low-resolution acquisition to avoid any kind of artifacts. The 3-D static volume box was placed over the highest villous vascular density zone at the insertion of the umbilical cord [27]. Each image was recovered from the disc in succession for processing. We recorded one sample from each patient during gestation.

### 4.4. Determination of Power Doppler Indices

The stored volumes were further analyzed using the virtual organ computer-aided analysis (VOCAL) program pertaining to the computer software 4DView (GE Medical Systems, Zipf, Austria, version 10.4) by the same expert in 3-D analysis (A.S.). The image used for recovering from the hard disc was captured and processed using a multiplanar system. The spherical sample volume was consistently 28 mL. The VOCAL program calculated the grey- and colour-scale values automatically from the acquired volume of spherical sample in a histogram in all cases. The combined use of power Doppler with three-dimensional ultrasound gives the possibility of quantifying blood in motion within a volume of interest. Three indices were calculated, namely the vascularization index (VI), flow index (FI), and vascularization flow index (VFI), as estimates of the percentage of volume filled with detectably moving blood. VI (expressed as a percentage) is the proportion of colour voxels in the studied volume, representing the proportion of blood vessels within the tissue. FI (expressed at a scale of 0–100) is the average value of all colour voxels, representing the average power Doppler amplitude within blood vessels. VFI (expressed at a scale of 0–100) is the average colour value of all grey and colour voxels and is the product of the number of colour voxels as a percentage and relative amplitude of these voxels [38,39]. The intra-observer errors were evaluated by repeated measurements of 3-DPD indices at initiation of the study. The intra-class correlation coefficients for all the Doppler indices were excellent (0.99) in the case of all indices.

The intra-observer errors were estimated by repeated measurements of 3-DPD indices at initiation of the study. The intra-class correlation coefficients for all the Doppler indices were excellent (0.99) in the case of all indices.

### 4.5. Procedure of Amniocentesis

The subjects were informed about the procedure and possible complications before the consent form was signed prior to the procedure. All the procedures were performed at the outpatient unit by the same operating expert (J.S.), who followed the standard protocol. Local antiseptics were applied to the skin. A 22-gauge spinal needle was inserted under continuous ultrasound guidance, and needle insertion through the placenta was avoided. Amniotic fluid (8–10 mL) was taken, and the first 2 mL of each sample was discarded to prevent contamination with maternal cells. Blood-contaminated amniotic fluid was not utilized. Fetal heart rate was determined after the procedure, and no stillbirth or premature rupture was observed. Following amniocentesis, anti-D immunoglobulin was administered, if it was necessary.

### 4.6. Samples

Amniotic fluid and maternal venous blood were collected from each subject at the time of amniocentesis. The blood samples were centrifuged at 3400 rpm for 15 min. The serum and amniotic fluid samples were stored at −80 °C until assay. The samples were retrieved from storage, thawed, and analyzed for laeverin and Gal-13.

### 4.7. Enzyme-Linked Immunosorbent Assay (ELISA)

Human Gal-13 and human laeverin in the maternal serum and amniotic fluid were determined by ELISA. The laboratory staff members who performed the assays were blinded to the pregnancy outcomes, and the clinician recruiting the women did not participate in analyzing the samples.

The concentration of Laeverin was measured using kits of Mybiosource (San Diego, CA, USA; Cat. No.: MBS2882930). The detection limit of the assay was 0.23 ng/mL, and the detection range was 0.312–20 ng/mL. The intra-assay coefficient was ≤4.7%, and the inter-assay coefficient was ≤6.3%, according to the manufacturer. 

The human Gal-13 levels were determined by Cusabio kits (Wuhan Huamei Biotec Co., Ltd., Wuhan, China). The sensitivity of the assay was <3.9 pg/mL. The intra- (<8%) and inter- (<10%) assay coefficients of variation were good according to the manufacturer.

### 4.8. Data and Statistical Analysis

The statistical analyses were performed using SPSS version 23 (IBM Corp., Armonk, NY, USA). Continuous variables were expressed as mean ± standard deviation (SD), and categorical variables were expressed as numbers and percentages. The relationship between the level of angiogenic factors (laeverin and Gal-13) and other continuous variables was assessed using Pearson’s correlation and regression analyses. The relationship between the level of laeverin and Gal-13, and other continuous variables was assessed using univariate and multivariate regression analyses interpreted by correlation coefficient (β) and (95% CI). Multiple linear regression was adjusted for well-known confounders such as for maternal age, BMI at amniocentesis, number of previous pregnancies, and gestational age at amniocentesis, as these factors determine the actual placental volume and fetal weight. Independent sample *t*-tests were used to determine whether the angiogenic factor levels in the body fluid were different in complicated pregnancies vs. subjects with no pregnancy complications. Associations between pregnancy outcome (hypertension-related diseases/GDM/fetal weight deviation vs uncomplicated pregnancies) and clinical characteristics of the participants and angiogenic factors measured in body fluids estimated by univariate and multivariate logistic regression are presented as odds ratios (ORs) with 95% confidence intervals (CIs). Fetal gender disparities in the serum and amniotic factor levels were tested by an independent samples *t*-test. The two-tailed statistical significance level was set at 5%, and the *p*-values were adjusted using a Holm–Bonferroni correction for multiple comparisons.

## Figures and Tables

**Table 1:** Clinical and obstetrical data of women with amniocentesis (N = 62).

Maternal age (years) *	34.53 ± 6.03
Number of nulliparous women in the study **	21 (33.9)
BMI at the time of genetical consultation (kg/m^2^) *	27.14 ± 6.03
Nuchal translucency at first trimester genetic sonography (mm)	1.97 ± 0.61
Gestational age at nuchal translucency (weeks)	12.75 ± 0.58
Gestational age at the time of amniocentesis (weeks) *	18.22 ± 1.35
Birthweight (grams) *	3406.29 ± 559.44
Gestational age at delivery (weeks) *	39.04 ± 1.41

* Continuous variables displayed as mean ± standard deviation (SD). ** Categorical variables are presented in number and %.

**Table 2:** Ultrasound data at amniocentesis (N = 62) *.

Fetal biometry	
Gestational age at the time of amniocentesis (weeks)	18.22 ± 1.35
Head circumference (mm)	151.02 ± 15.47
Head circumference (percentile)	51.23 ± 29.62
Abdominal circumference (mm)	127.86 ± 15.65
Abdominal circumference (percentile)	49.44 ± 28.30
Femur length (mm)	26.90 ± 4.70
Femur length (percentile)	54.66 ± 31.37
Estimated Birthweight (grams)	238.69 ± 69.82
Estimated Birthweight (percentile)	50.93 ± 26.62
Placental sonography	
Placental volume (mm^3^)	230.38 ± 93.96
VI	13.90 ± 5.09
FI	45.44 ± 24.02
VFI	7.77 ± 3.43

VI: Vascularization Index, FI: Flow Index, VFI: Vascularization Flow Index. * Continuous variables displayed as mean ± standard deviation (SD).

**Table 3:** Levels of angiogenic factors in samples of serum and amniotic fluid (N = 62) *.

Gal-13 concentration in serum (pg/mL)	204.23 ± 171.34
Gal-13 concentration in amniotic fluid (pg/mL)	8.68 ± 9.85
Laeverin concentration in serum (ng/mL)	0.93 ± 0.74
Laeverin in amniotic fluid (ng/mL)	14.11 ± 9.18

Gal-13: Galectin-13. * Continuous variables displayed as mean ± standard deviation (SD).

**Table 4:** Correlation between maternal as well as sonographic data and the levels of angiogenic factors in the maternal serum and amniotic fluid (N = 62).

	**Laeverin in Serum**				**Laeverin in Amniotic Fluid**			
	**Univariate Linear Regression**		**Multivariate Linear Regression**		**Univariate Linear Regression**		**Multivariate Linear Regression**	
	**β**	**CI**	**β**	**CI**	**β**	**CI**	**β**	**CI**
Gal-13 in serum	−0.38 *	−0.01–−0.01 *	−0.31	−0.01–0.01	−0.15	−0.06–0.02	−0.11	−0.06–0.03
Gal-13 in amniotic fluid	−0.32 *	−0.04–0.01 *	−0.36	−0.06–0.01	−0.13	−0.44–0.19	−0.16	−0.53–0.23
Laeverin in serum	-	-	-	-	0.03	−3.59–4.26	−0.01	−4.29–4.20
**Gal-13 in Serum**					**Gal-13 in Amniotic Fluid**			
	**Univariate Linear Regression**		**Multivariate Linear Regression**		**Univariate Linear Regression**		**Multivariate Linear Regression**	
	**β**	**CI**	**β**	**CI**	**β**	**CI**	**β**	**CI**
Gal-13 in serum	-	-	-	-	0.04	−0.01–0.02	−0.11	−0.06–0.03

Gal-13: Galectin-13, * *p* < 0.05.

**Table 5:** Correlation between maternal as well as sonographic data and the levels of laeverin in the maternal serum and amniotic fluid (N = 62).

	Laeverin Serum Level				Laeverin in Amniotic Fluid			
	Univariate Linear Regression		Multivariate Linear Regression		Univariate Linear Regression		Multivariate Linear Regression	
	β	CI	β	CI	β	CI	β	CI
Clinical and obstetrical characteristics								
Maternal age	−0.34 *	−0.06–0.01 *	−0.15	−0.06–0.03	−0.11	−0.63–0.31	−0.14	−0.47–0.19
Previous parity	−0.04	−0.29–0.22	0.03	−0.26–0.32	0.01	−3.13–3.31	0.01	−3.62–3.72
BMI at the time of genetical consultation (kg/m^2^)	−0.19	−0.06–0.01	−0.14	−0.05–0.02	−0.18	−0.71–0.19	−0.18	−0.75–0.23
Birthweight (grams)	−0.01	0.01–0.01	0.02	0.01–0.01	0.01	−0.01–0.01	0.01	−0.01–0.01
Birthweight (percentile)	−0.19	−0.01–0.01	−0.15	−0.01–0.01	−0.03	−0.11–0.09	−0.03	−0.11–0.09
GA at delivery (weeks)	0.39 *	0.01–0.07 *	0.41 *	0.01–0.07 *	0.04	−0.36–0.47	−0.15	−0.47–0.19
GA at the time of amniocentesis (weeks)	0.24	−0.01–0.04	0.17	−0.01–0.04	−0.06	−0.35–0.24	−0.15	−0.47–0.19
Fetal sonography at the time of amniocentesis								
Head circumference (mm)	0.18	−0.01–0.02	−0.21	−0.04–0.02	0.03	−0.17–0.21	−0.03	−0.23–0.19
Head circumference (percentile)	−0.32 *	−0.01–0.01 *	−0.15	−0.01–0.01	0.19	−0.04–0.16	0.21	−0.04–0.17
Abdominal circumference (mm)	0.15	−0.02–0.03	−0.26	−0.08–0.05	0.55 *	0.02–0.56 **	0.11	−0.18–0.27
Abdominal circumference (percentile)	−0.08	−0.02–0.03	−0.05	−0.02–0.02	−0.19	−0.18–0.08	−0.18	−0.16–0.07
Femur length (mm)	0.24	−0.02–0.09	−0.12	−0.16–0.13	0.20	−0.23–0.76	−0.11	−0.66–0.38
Femur length (percentile)	−0.07	−0.01–0.01	−0.07	−0.01–0.01	0.29	−0.02–0.15	0.16	−0.04–0.11
Estimated fetal weight (grams)	0.15	−0.01–0.01	−0.51	−0.03–0.02	0.48 *	0.01–0.68 *	0.22	−0.03–0.07
Estimated fetal weight (percentile)	−0.14	−0.02–0.01	−0.08	−0.02–0.02	0.09	−0.11–0.16	0.04	−0.12–0.14
Placental sonography at the time of amniocentesis								
Placental volume (mm^3^)	0.06	−0.01–0.01	0.17	−0.01–0.01	0.32 *	0.01–0.46 *	0.42	0.01–0.07
VI	0.07	−0.04–0.05	0.12	−0.03–0.06	0.05	−0.47–0.63	0.05	−0.51–0.69
FI	−0.14	−0.01–0.01	−0.14	−0.01–0.01	0.01	−0.10–0.10	−0.01	−0.11–0.11
VFI	0.05	−0.06–0.08	0.12	−0.05–0.10	−0.06	−1.06–0.74	−0.03	−1.05–0.86

BMI: body mass index, GA: Gestational age, VI: Vascularization Index, FI: Flow Index, VFI: Vascularization Flow Index. * *p* < 0.05, ** *p* < 0.001.

## Data Availability

The data can be made available by the corresponding authors on request.
